# New rules for genomics-informed COVID-19 responses–Lessons learned from the first waves of the Omicron variant in Australia

**DOI:** 10.1371/journal.pgen.1010415

**Published:** 2022-10-13

**Authors:** Ashleigh F. Porter, Norelle Sherry, Patiyan Andersson, Sandra A. Johnson, Sebastian Duchene, Benjamin P. Howden

**Affiliations:** 1 Department of Microbiology and Immunology, The University of Melbourne at The Peter Doherty Institute for Infection and Immunity, Melbourne, Australia; 2 Microbiological Diagnostic Unit Public Health Laboratory, The University of Melbourne at The Peter Doherty Institute for Infection and Immunity, Melbourne, Australia; CWRU: Case Western Reserve University, UNITED STATES

During the COVID-19 pandemic, phylodynamics and phylogeography have been launched into the spotlight as tools to model the spread of the SARS-CoV-2 virus. In Australia, we have relied on genomic epidemiology (and associated derived parameters such as viral growth rate, reproductive number, and estimated sampling proportion) to inform public health policy changes [1]. This was possible due to the high proportion of SARS-CoV-2 cases sequenced in Australia throughout 2020 and 2021, where we maintained low burdens of both cases and deaths. The recent Omicron ‘waves’ experienced in Australia and globally, combined with the relaxation of public health restrictions, has seen a significant jump in Australia’s case numbers, rising to the top 10 globally in newly reported cases and deaths in August and September 2022 (https://covid19.who.int/table). With around 10-fold more cases per day in 2022 compared to the previous year, our sequencing strategy has had to adapt along with the virus. Here, we emphasise how in the ‘COVID-normal’ future, the way we sequence during high-case load settings can optimise our application of phylogenomic methods to sufficiently inform the COVID-19 pandemic response.

## The rise of the Omicron variant

**Fig 1:**
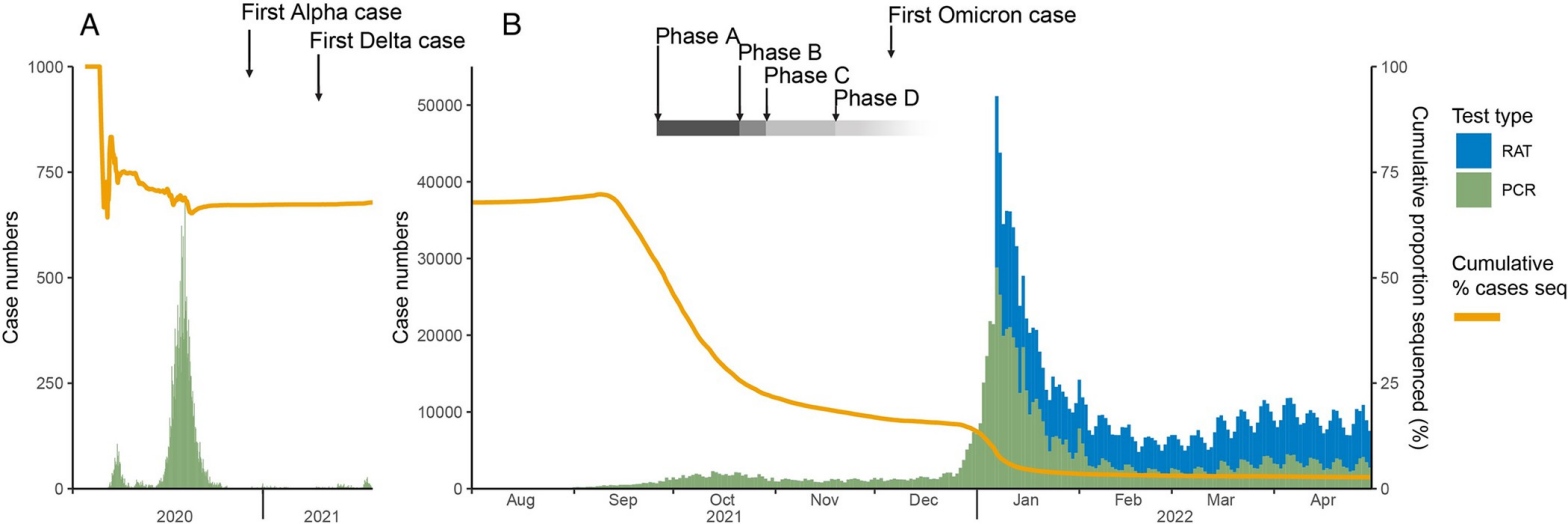
Epidemiological curve of SARS-CoV-2 in Australia, demonstrating the rapid rise in cases in 2022 upon emergence of the Omicron variant, and the diminishing cumulative proportion of cases sequenced. Panel A has a reduced scale to visualise the limited case numbers during January 2020 to July 2021 inclusive. Panel B includes data from August 2021 to April 2022 inclusive. The case numbers (Y-axis, left) obtained from PCR tests and rapid antigen tests (RAT) are respectively shown in green and blue. The cumulative percentage of cases sequenced (Y-axis, right) is visualised as an orange line. The emergence of variants of concern (Alpha, Delta and Omicron), as well as the 4 phases of the Victorian Government’s “Roadmap” [2] for easing COVID-19 restrictions, are shown at the top of the curve. The Victorian Roadmap is an example of jurisdiction-managed responses to the COVID-19 pandemic. The roadmap included four phases (A-D) that gradually eased restrictions on travel and social distancing measures, partly based on the percentage of the population fully vaccinated. Phase A focused on returning students to the classroom, whereas Phase B and C guided the return to work and travel upon reaching vaccination targets of 70% and 80% of the population (16+) fully vaccinated, respectively. The final stage, Phase D, was reached when 80% of the population (12+) was fully vaccinated, allowing the restrictions to ease and align with Australia’s National COVID-19 response.

During the first stage of the pandemic (January 2020 to November 2021), a comprehensive genomics-informed response was possible in Australia, due to the low case numbers of COVID-19. The low case numbers also enabled one of the highest genome sequencing rates globally (up to 80% of Victorian cases sequenced in the “second wave” in 2020, Fig 1) [1]. However, the introduction of the Omicron variant in late 2021, coinciding with the lifting of restrictions, led to an exponential increase in cases (Fig 1). We anticipate there was a significant number of undiagnosed cases (e.g. PCR tests underestimating true transmission [3]), during the peak of the Omicron wave, due to the overwhelming demand for testing, which inundated the established diagnostic PCR testing systems and created a shortage of rapid antigen tests. Furthermore, half of the reported COVID-19 cases were being counted from positive rapid antigen tests (Fig 1), which were not able to be sequenced. The ongoing Omicron wave has had significant local and global impacts, underlining the necessity to prepare for future variants, including Omicron subvariants and other emerging variants of concern (VOCs) that are not manageable by current public health control methods.

Considering this, we discuss the ripple effect of the Omicron wave in Australia, as well as which sequencing strategies will be most effective for modelling the COVID-19 pandemic, both locally and globally. We highlight Australia’s focus on genomic epidemiology, a field focused on understanding the spread of a pathogen through a population by combining genomic data and epidemiological metadata. Specifically, we discuss phylodynamics, which utilises epidemiological and genomic data to explore how evolution and epidemiology drive phylogenetic patterns [4], and phylogeography, which focuses on the processes that determine the spatial distribution and spread of observed lineages [5]. Although estimates from these methods have had a large impact on pandemic response, there are three major issues Australia (and the rest of the world) will be facing:

- We cannot sustain our previous rates of sequencing, especially with highly transmissible VOCs and elevated case numbers.
- Even if we could sequence every positive case, our models are unable to incorporate the full Australian dataset, let alone global sequence data.
- Genome surveillance alone may not be sufficiently informative to produce meaningful epidemiological estimates.

Therefore, we argue here that *more genomes* do not necessarily mean *better results*.

As there has never been such an extensive pathogen genomics dataset (and such a high degree of public interest) to fully utilise genomic epidemiological methods, our major question for our long-term COVID-normal future is: how sustainable will it be to continue to generate this data, and more importantly, is it beneficial for the global response?

## Sustainable sequencing to track the COVID-19 pandemic

Whilst public health and social measures, quarantine restrictions and vaccination have all been utilised in past and current pandemics, the COVID-19 pandemic is the first to employ genomic sequencing on a massive global scale. Genomics has provided a major advantage to pandemic control by improving our understanding of the underlying transmission dynamics and evolution of SARS-CoV-2 in near-to-real time. Australia’s initial approach to COVID-19 management involved strict non-pharmacological interventions (border closures, travel restrictions and social distancing measures) which resulted in elimination of the virus in Australia for several months between 2020 and 2021 (Fig 1). This tactic was coupled with coordinated diagnostic testing and sequencing efforts, resulting in approximately 50% of known cases nationally having a sequenced genome. In both Australia and New Zealand, where the proportion of cases sequenced has been substantial, we have been able to use the data at the “macro” level (studying global evolution of the virus, emergence of VOCs and informing public health policies) and at the “micro” level (inferring local transmission networks and the impact of public health interventions on genomic clusters) [1,6–9]. For example, genome data unequivocally traced most cases from a large outbreak in Victoria to a single hotel quarantine breach [1], leading to major policy changes in how Australia manages hotel quarantine facilities. Amendments to hotel quarantine included improvements to training (specifically for infection control practices), restriction of employment (to reduce community spread from employees working multiple jobs) and increased testing of employees. Furthermore, the impact of these changes can be observed, with a vast improvement to hotel quarantine escape risk in the period after policy change [1].

However, upon the easing of restrictions in late 2021 [1], the rapid spread of Omicron quickly overloaded the established testing capability and stretched the capacity of healthcare services. Due to the rapid rise in case numbers, the SARS-CoV-2 sequencing proportion was markedly reduced (Fig 1). Although a high-level national sequencing strategy was rapidly developed for Australia, the plan was dedicated to managing the public health response. The strategy aimed to balance sequencing of priority groups (determined by public health requirements) with surveillance of community infections, but implementation varied between jurisdictions. This was due to variable capacities, logistics and pandemic stage within jurisdictions (for example, one state did not experience community transmission until many months after the other jurisdictions).

In reconsidering our sequencing strategies and looking forward, we believe that the sequencing strategy could be further optimised from a modelling perspective to utilise our resources effectively [10]. Attempting to sequence the previously high proportion of cases is now unfeasible, and furthermore, we have found that “inconsequential sequences” (such as closely related sequences from a household-wide infection) provide diminishing returns for guiding the response. When looking towards the future, we will require a system that will sustainably sequence a proportion of positive COVID-19 cases during periods of high case numbers. Importantly, our strategy also needs to balance background, community-level “representative” sequencing along with “focused” sequencing (e.g. returning travellers), to ensure we are gathering the full diversity of lineages as new VOCs continue to emerge and spread (as seen with the sub-variants of the Omicron variant). We also note that SARS-CoV-2 sequencing strategies should *continue* to evolve as case numbers and the public health landscape changes, hence any sequencing strategies should continue to be re-evaluated during their implementation.

## COVID-19 and the new era of genomic epidemiology

The COVID-19 pandemic has caused a major shift in the field of genomic epidemiology and surveillance, as reviewed previously [11–13]. In the last decade, increased computational power has enabled scientists to analyse large and complex viral datasets, such as HIV, Ebola, influenza and Zika viruses [14–16]. However, the volume of genomic data generated for the COVID-19 pandemic has vastly outstripped past epidemics—for example, the current count of SARS-CoV-2 genomes is at least five orders of magnitude greater than the sequencing effort for decades-long history of past outbreaks, such as swine flu. Although complex phylodynamic models have been developed before the COVID-19 pandemic (such as those developed for Ebola surveillance) [17], these methods have not previously been applied to such a sizeable dataset.

The power of applying genomic epidemiological models to the SARS-CoV-2 dataset has recently been thoroughly reviewed [18], demonstrating how the combination of genomic data with epidemiological metadata, such as travel history [19] or healthcare associated infections [20], has allowed us to prepare appropriate outbreak responses in real-time. Phylogenetic modelling has been used to explore the transmission, spatial dispersal and epidemiology of SARS-CoV-2 [21], for example; it has been used to distinguish community transmission from novel importations in China [22], the emergence and spread of new variants in the USA and South Africa [23–25], the impact of public health interventions on emerging lineages in Brazil [26], and detailed outbreak transmission dynamics across Australia and New Zealand [1,6,7,9]. However, there are several major issues of applying these models to the SARS-CoV-2 global sequence dataset.

### An overwhelmingly large and complicated dataset

Firstly, our models are computationally unable to incorporate all the sequence data available. This is complicated by a connected issue, the quality of the data. Although this is not unique to the SARS-CoV-2 dataset, data gathered from large public databases (such as GISAID) is notoriously poorly formatted (with formatting inconsistency and errors, along with missing metadata) and can range dramatically in sequence quality (i.e. large gaps that create issues in comparative analyses). All these factors can complicate downstream analysis, contributing to the infamous “bioinformatics bottleneck” [27].

### Widespread sampling bias throughout dataset

There has been a noticeable bias in SARS-CoV-2 sampling, especially spatiotemporal bias [18,27,28]. The bulk of genomes sequenced are sourced from the UK, Europe and North America, which account for almost 90% of all genomes in GISAID. This sampling bias contributes to the difficulty of drawing comparisons from different models and spatiotemporal datasets [28]. Presently, there are no guidelines available for utilising the immense (and sometimes overwhelming) dataset, however, commonly used approaches to attempt to reduce sampling bias include subsampling the available genomes geographically and temporally [26] or using global diversity via the Nextstrain “backbone” [29].

The sampling bias present in the SARS-CoV-2 dataset is incompatible with most phylogeographic methods, including discrete trait ancestral reconstruction [30]. However, this has led to innovative solutions—the integration of additional information such as individual travel history, transportation data and epidemiological data into extended phylogeographic models. Importantly, the inclusion of this new information suggested alternative hypotheses not apparent using only genomic and geographic data [18]. Although these novel methods have proven powerful, the availability of the necessary metadata is not consistent–for example, even if case travel history metadata is collected, it is not shared on public databases.

### Incompatibility between datasets

Finally, although it is tempting to explore the spatiotemporal spread of SARS-CoV-2, several complications arise when trying to review the “global” findings. Not only is the epidemiological data dependent on each location (e.g. population size and structure), but the sampling proportion varies dramatically between countries. As an example of two countries using different approaches, teams from Brazil and the USA both combined genomic epidemiology with two different types of movement data (travel pattern analysis and flight data) [31,32]. However, the dataset that was used in these models would be biased by the fact that North America had a much higher sampling proportion (1.5%) compared to South America (0.3%). Also, critically, the location where the COVID-19 case was sequenced does not equate to location where it was acquired (a variable which would benefit greatly from collection and storage of travel history metadata on sequence-sharing platforms).

Generating results from different datasets with the same model does not necessarily mean we can draw comparisons—for example, it is not reasonable to contrast the reproductive number of the same variant (e.g. Omicron) circulating in two different locations, as differences in population dynamics (including population size, density, vaccination status, susceptibility due to previous VOC infection, and social distancing measures) will dramatically bias results. However, we can reduce this bias with thorough metadata collection (i.e. vaccination coverage). Ideally, each dataset requires a unique approach designed specifically for the hypothesis being tested, particularly for methodologies that involve a variety of elements, such as phylogeography. When looking forward, novel tools include the use of negative controls in discrete phylogeographic models [33], and the potential to use both sequence data and “non-sequenced” data (positive cases that did not undergo whole genome sequencing) [34].

## How do we collect our “ideal” dataset?

**Fig 2:**
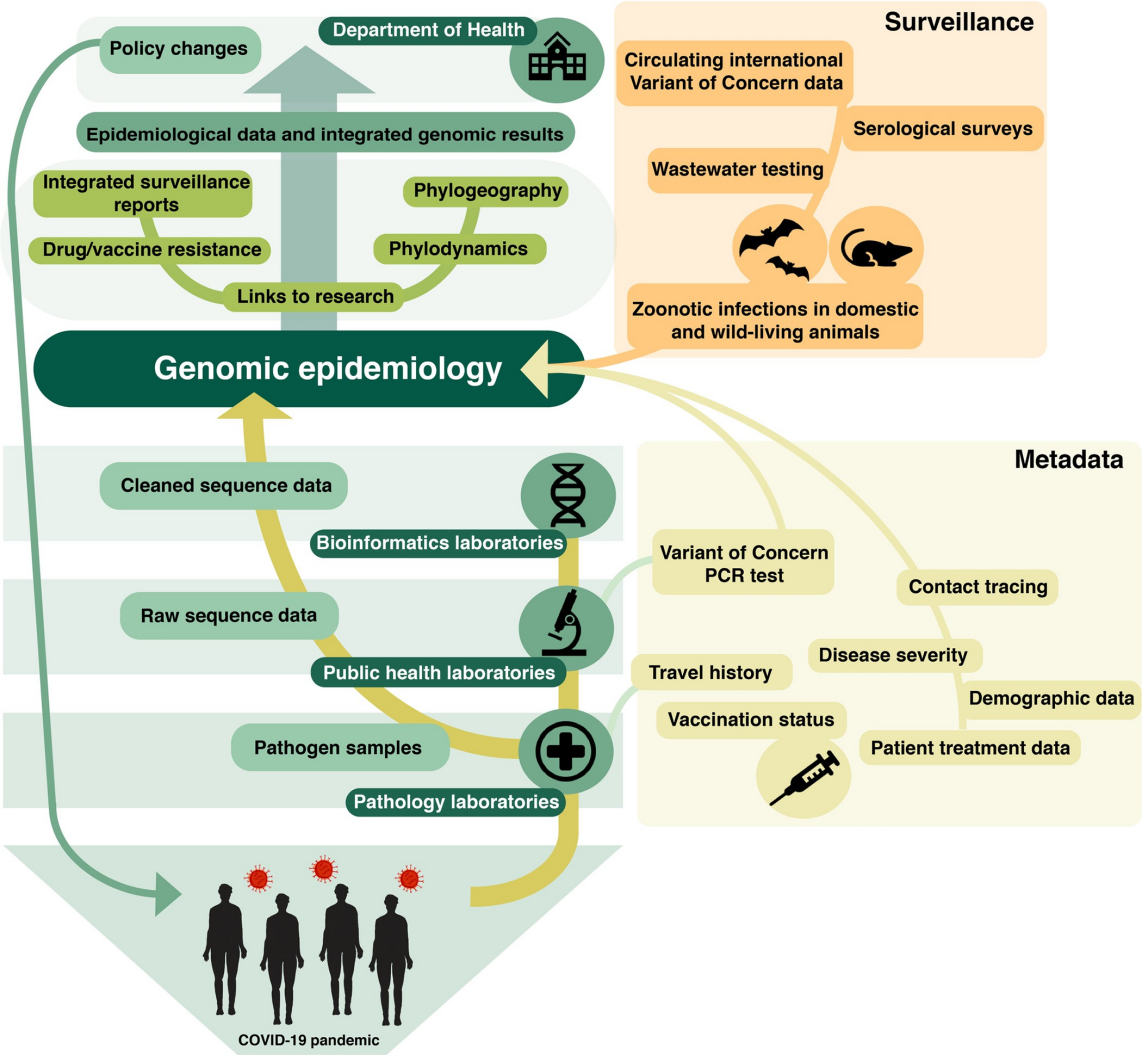
Visualisation of different sources of COVID-19 data, including sequence data and metadata, and how they feed into genomic epidemiology and link to research and policy making decisions. Here we highlight the flow and collaboration within the Australian COVID-19 pandemic response network, between public and private pathology, public and private pathology laboratories, bioinformatics laboratories, research groups and the health departments. The infographic on the left represents the pipeline of sequence data, sourced from samples collected from the public, feeding into genomic epidemiology and phylogenomic tools. The coloured bubbles on the right represent additional sources of data, such as epidemiological metadata (yellow bubbles) or global surveillance data (orange bubbles).

**Fig 3:**
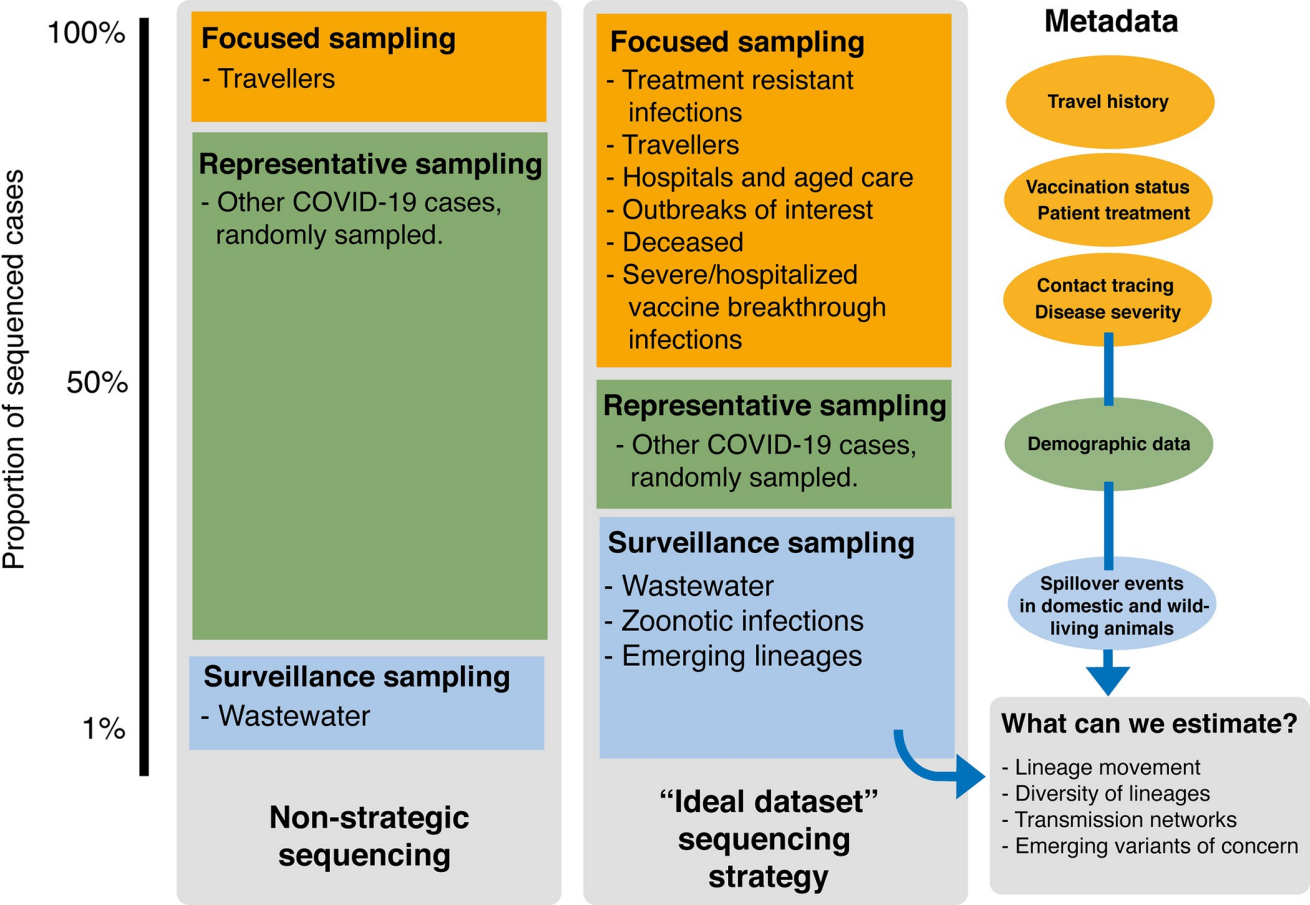
Illustration of the current “non-strategic” sequencing used for monitoring the COVID-19 pandemic, in comparison to the plan we have outlined as our “ideal dataset”. The two main streams of sequencing we have described, focused and representative sampling, are represented by the orange and green boxes, respectively. These streams are shown along with surveillance sampling, represented by the blue box. The size of each box represents the proportion of sequenced genomes being generated from each stream. The associated metadata with each stream is represented by a coloured bubble in the right panel. Following the blue arrows, we overview the parameters we can estimate from the “ideal dataset” sequencing strategy, combined with metadata.

As we have hinted at previously, even if we committed to collecting every sequence of a COVID-19 infection, the advantages gained from high-volume sequence data would presently not equal the investment. However, if we focused our resources towards collecting high quality data–both a sufficient level of “representative” and “focused” sequencing, and comprehensive metadata (Fig 2), this is where we might see real benefits to our downstream analysis. Based on a modelling perspective, we require a minimum of 10–100 sequences to inform a phylogenetic tree. To inform public health management of the COVID-19 pandemic, 10% of cases is sufficient to obtain meaningful phylogenetic resolution (due to the lack of genetic diversity within SARS-CoV-2 lineages). However, in high case-load scenarios (such as those we have seen in the Omicron wave), where it would be unreasonable to sequence such a high proportion of cases, we recommend focusing on representative sequencing of a smaller percentage of cases (1–2%) [35] (Fig 3).

With this dataset, we could extend past the basic genomic epidemiological methods and focus on the outputs from complex, informative phylodynamic and phylogeographic models. As seen in Fig 2, there are many other sources of data that can be gathered to benefit genomic epidemiology, such as patient metadata (vaccination status, treatment history). Additionally, we could benefit from early detection of outbreaks by setting up continuous surveillance systems, such as wastewater surveillance, serological surveys, and sampling zoonotic spillover events in wildlife, livestock and domestic animals. Not only will including these streams of data into our models reduce the necessity to sequence a large proportion of COVID-19 cases, but evidence suggests that combining sequence data and metadata could be more informative for informing public health measures [19,36].

Additionally, although there has been much work on COVID-19 research, one area of improvement we could all benefit from is *connections*. There are several elements involved in making progress towards a COVID-normal future (overviewed in Fig 2). Examples include breaking down barriers between organisations, building links between research and public health groups, establishing governance mechanisms to facilitate data distribution, establishing effective communication between disciplines, and forming channels to share data [17]. Importantly, building these multidisciplinary connections would not come at a major cost–instead, we can build on the processes and platforms that have already been developed, which have been supported by investments that have already been made for managing the COVID-19 pandemic.

## Future directions for genomics-informed surveillance of COVID-19 and imminent pandemics

As we anticipate future “waves” to occur with the emergence of novel VOCs (as part of the continued evolution of SARS-CoV-2) we need to develop proactive strategies to optimally use genomics for managing the COVID-19 pandemic. Emerging VOCs may demonstrate increased transmissibility and immune evasion, including vaccine breakthrough as seen with the Omicron variant [37,38], presenting challenges for tracking cases and maintaining sequencing levels. New challenges are constantly developing, such as Omicron subvariants BA.2, BA.4 and BA.5 evolving the capability to re-infect despite vaccination or previous SARS-CoV-2 infection [39], and the rise of SARS-CoV-2 Omicron recombinants (a result of recombination via co-infection with multiple lineages) [40]. Furthermore, with global movement now returning to normal levels, nations must prepare for the circulation of a diversity of SARS-CoV-2 lineages and emerging VOCs, as well as multiple introductions from global locations. To dedicate our resources towards capturing the diversity of lineages, identifying VOCs, and rapidly detecting new outbreaks, we need an effective plan to strategically gather data (both genomic sequencing and metadata, as seen in Fig 2).

Hubs and other organisational structures that support continual genomic sequencing for SARS-CoV-2 surveillance have been beneficial during the pandemic (as seen with UKHSA), and moving forward, supporting equitable access across the globe should be a priority. These “hubs” should include multidisciplinary teams, including scientists with backgrounds in microbiology, molecular biology, epidemiology and phylogenetics, as well as clinicians and public health experts. Although sequencing and analyses are performed in each Australian jurisdiction, a centralised platform has been established to share national sequences and limited metadata (e.g. quarantine status, travel history), termed AusTrakka [41]. This platform has proven especially useful for managing SARS-CoV-2 surveillance across Australia. Other countries could benefit from utilising this model locally, but we could all benefit from applying this model on a global scale. Importantly, these hubs would support equitable access to genomic sequencing, which is crucial for countries with limited resources.

Given the increasing complexity of the SARS-CoV-2 landscape and high case numbers, it is critical to establish and maintain consistent surveillance of SARS-CoV-2 on multiple fronts, including community sampling and wastewater detection. We should not ignore the potential for spillover and spillback events, along with the formation of viral reservoirs, in wild-living and domesticated animals. We emphasise a one health approach (and teams that reflect veterinary, epidemiological, and ecological knowledge) for surveillance of spillover events and potential reservoirs in animals (e.g. minks, mice and deer) [42–44]. In Australia, our wastewater surveillance system is at the forefront for early detection lineages or variants of concern, as seen with the identification of Omicron sub-variants BA.2, BA.4 and BA.5 in wastewater [45,46], alerting authorities to be on the lookout for a rise in cases and contributing to modelling to inform public health.

For surveillance of clinical samples, we need to consider the aims of sequencing in each setting to inform the local sequencing strategy, and utilise our resources most effectively for measuring the evolution and spread of the virus. In this current phase in Australia, SARS-CoV-2 sequencing primarily focuses on determining proportions of current variants, identifying new emerging variants, and identifying the introduction of new VOCs into the community. Part of the current strategy includes gathering data on disease severity to inform public health activities, including modelling. Secondary aims may include assessing the performance of diagnostic tests and drug therapies with new variants, and investigation of specific outbreaks or populations (e.g. healthcare-associated outbreaks or prolonged infections in immunosuppressed cases). We note that there is an inherent trade-off in the choice to focus on target populations versus the general community. Whilst over-representation of cases with severe disease provides more data about disease severity and healthcare utilisation, this focus reduces the sensitivity of detecting the emergence or introduction of new variants in the community, hence delaying the time to identification of these critical events.

To achieve the aims listed above, sequencing should ideally be continual, rather than sporadic, as “sequencing blitzes” only gather information from closely related sequences and provide no temporal overview. Instead, community-based genomic surveillance provides consistent coverage of SARS-CoV-2 evolution–both temporally and geographically [47]. Along with continual, strategic sequencing (overviewed in Fig 3), the collection of basic metadata is essential to the practical use of COVID-19 data, including sample collection date, symptom onset date, exposure site history, travel history, vaccination status, and previous COVID-19 history. This collection strategy will provide valuable information, (especially if shared on a data platform such as AusTrakka), particularly for outbreaks of interest (i.e. to focus on a hospital-associated outbreak).

We recommend two main streams of sequencing [10]:

1. Focused sampling: Confirmed cases (positive nucleic acid amplification tests) from target groups of interest (outbreaks, travellers, hospitalizations, aged care, deceased, severe/hospitalized vaccine breakthrough infections): sequence as much as possible and capture essential metadata on travel history, vaccination status, and/or hospital history.
2. Representative sampling: Confirmed cases (positive nucleic acid amplification tests): sequence randomly and collect relevant metadata to contextualise the background prevalence of genomic lineages circulating.

These two streams of sequencing will need to be combined with consistent surveillance (e.g. wastewater and zoonotic spillovers) and a strategy for collection of essential metadata (Fig 3). Furthermore, we recommend a central platform for integrated pathogen genomics epidemiology, exemplified by the use of AusTrakka in Australia [41].

A COVID-normal approach will require public health and research teams to focus on the emergence and transmission dynamics of SARS-CoV-2 lineages, along with associations to COVID-19 disease severity. Importantly, genomic epidemiology can assist with understanding the emergence and evolution of SARS-CoV-2 and transmission dynamics if sufficient sequence data and metadata is available. For example, as shown in Fig 3, past strategies for SARS-CoV-2 sequencing around the globe have closely resembled the left box, where most SARS-CoV-2 genomes have been collected from positive COVID-19 cases indiscriminately (excluding incoming travellers). Although there has been considerable genomic data collected in Australia, this approach, in conjunction with limited metadata, has hindered our ability to draw meaningful conclusions from the dataset as case numbers rise. However, we propose the application of the streams of sequencing outlined above, which would provide sufficient information to estimate many important parameters, even in scenarios where COVID-19 case-loads are high (Fig 3).

We can also supplement samples that were unable to be sequenced with supporting information, such as metadata (i.e. results from a VOC PCR test), to inform downstream analysis (Fig 2). With this strategy, we are confident that despite sequencing a smaller proportion of overall COVID-19 cases, the additional streams metadata will provide sufficient information to draw informative estimates (Fig 3).

Much of the long-term COVID-normal future will be informed by our ability to exploit genomic epidemiology through gathering data about SARS-CoV-2 (both at the sequence and metadata level) and *sharing* it. We believe a global, coordinated response for data collection and modelling will be essential, both for the ongoing COVID-19 pandemic and future infectious disease outbreaks.
